# Failed

**Published:** 1889-01-12

**Authors:** A. H. Begbie


### "FAILED."

I.

"I have failed," he said, "I have failed, even where I thought to excel,
The book must fall from my hand an' I scarce have learned to spell,
And my tongue has lost the message God breathed on my soul to tell."

II.

"I have lost it," he said, "and what in its place will you say I have won?
What is the priceless treasure I staked my soul upon?
Look! this poor painted fruit, with its cold, hard heart of stone."

III.

"I dreamt to climb," he said, "climb the white hill of God;
But, alas! I rested my soul on the earth's soft emerald sod,
Where the winged foot of an angel scarce durst for a moment have trod.

IV.

"The tree of life grew back—back from the shining hill;
They said 'Eat, it is good for a man; eat and take thy fill;'
And then I stretched out my hand, till its trembling at last grew still.

V.

"And I plucked me this earth-apple, I plucked this death-tainted fruit,
With the canker-worm that gnaws, unceasingly gnaws at the root,
Till, at last, my soul sank untuned, as it were a dumb, stringless lute.

VI.

"And, look you, this makes Hell—loathing sin's dark defile,
Sinning, for ever sinning, with yet a fierce hunger the while,
To creep from God's desolate frown into the light of His smile.

VII.

"Oh, God! to have dreamt of Thy Heaven, of the light of Thy face, to know
Of that marble Temple of Truth, in whose garden the white lilies blow,
And then with this vision before me to fall for ever below."

VIII.

He paused—Time's shuddering waters silently ebb and swell,
The blood-red tears of a soul in tune to their surging fell—
Of a soul that was plumed for Heaven, and seemed to be lost in a Hell.

IX.

Lost! nay not wholly lost! If the Vision Divine remain
The Christ of God is strong to bid thee turn again,
Then rise, oh trembling soul! by His steps deep carv'd of Pain.

—*A. H. Begbie.*

